# Systematic review of different approaches for performance enhancement in elite sport

**DOI:** 10.3389/frai.2026.1781958

**Published:** 2026-03-09

**Authors:** Oualid Dehbane, Sara Ouahabi, Sanaa El Filali

**Affiliations:** Faculty of Sciences Ben M'Sick, Hassan II University, Casablanca, Morocco

**Keywords:** artificial intelligence, deep learning, elite sport, injury prevention, machine learning

## Abstract

**Background:**

Elite sport is undergoing rapid technological transformation driven by advanced analytics, artificial intelligence (AI), and immersive systems. While numerous studies address performance enhancement and injury-related applications, evidence remains fragmented across technologies and sport contexts.

**Objective:**

This systematic review aimed to examine the prevalence and distribution of advanced analytical technologies across application domains (performance, injury, and emerging objectives) and sport disciplines, and to identify areas of technological maturity in elite sport.

**Methods:**

A systematic review was conducted following PRISMA 2020 guidelines. Four databases (Google Scholar, Scopus, Web of Science, IEEE Xplore) were searched for peer-reviewed studies published between January 2019 and March 2025. Fifty-two studies met the inclusion criteria and were synthesised using a structured qualitative approach.

**Results:**

AI-based methods dominated the literature (32/52 studies, 61.5%), including machine learning (15.4%), deep learning (9.6%), generative AI (17.3%), and hybrid approaches (19.2%). Statistical modelling accounted for 23.1% of studies, while virtual reality represented 15.4%. Performance enhancement was the primary objective (52%), followed by injury-related outcomes (27%) and emerging applications such as tactical analysis and decision support (21%). Team sports, particularly football, demonstrated the highest level of technological maturity.

**Conclusion:**

Advanced analytical technologies are unevenly distributed across sport disciplines and objectives, with clear maturity in performance-focused team sport applications. These findings provide evidence-based guidance for researchers and practitioners seeking to prioritise effective and context-appropriate technological adoption in elite sport.

## Introduction

Elite sport is entering a phase of accelerated technological transformation driven by the convergence of large-scale data availability, increased computational capacity, and rapid advances in intelligent and immersive systems. Performance enhancement and injury management—longstanding priorities in high-performance sport—are increasingly addressed through analytical technologies designed to optimise decision-making, personalise training processes, and support athlete health across competitive lifecycles (Cossich et al., 2023; Baladaniya and Choudhary, 2025). Advanced statistical modelling, machine learning (ML), deep learning (DL), virtual and augmented reality (VR/AR), and, more recently, generative artificial intelligence (GenAI) are progressively reshaping how performance is measured, interpreted, and acted upon in elite contexts.

Until recently, analytical practice in elite sport relied predominantly on descriptive and inferential statistical approaches combined with expert judgement. Since the late 2010s, however, predictive and prescriptive models capable of processing high-dimensional and multimodal data have become increasingly prominent (Van (Van Eetvelde et al., 2021)). In parallel, VR technologies have enabled controlled yet ecologically valid environments for skill acquisition, perceptual–cognitive training, and rehabilitation (Edwards and Hettinga, 2023). From 2023 onward, the emergence of GenAI—including large language models and generative simulation systems—has introduced new capabilities such as automated tactical scenario generation, personalised training content, and natural language interaction between coaches, athletes, and analytical tools (Puce et al., 2025; Baughman et al., 2024). These developments collectively signal a shift from isolated analytical tools toward integrated socio-technical systems embedded within performance and medical decision-making.

Despite the rapid expansion of this research domain, the existing literature remains fragmented. Previous reviews have typically focused on single technologies or narrowly defined applications, such as ML-based injury prediction (Zouhal et al., 2021), VR-based training (Zhao and Xueying Guo, 2022), or optical tracking systems (Naik et al., 2022). While informative, these reviews rarely compare technologies across application domains, seldom integrate GenAI within the broader performance ecosystem, and provide limited critical assessment of methodological robustness, ethical constraints, or organisational adoption. As a result, there is currently no comprehensive synthesis examining how advanced analytical technologies are distributed across performance, injury, and emerging objectives, nor how their growing influence reshapes human expert judgement in elite sport.

The period from 2019 to 2025 represents a critical window for such an analysis. This timeframe captures the transition from classical statistical approaches toward AI-driven systems, the proliferation of wearable and video-based data streams, and the rapid maturation of GenAI technologies after 2023. Importantly, it also reflects a shift from proof-of-concept research toward applied systems deployed in high-stakes tactical and medical environments, raising new concerns related to bias, interpretability, governance, and over-reliance on algorithmic outputs (Rahimian and Toka, 2022; Jud and Thalmann, 2024).

Accordingly, this systematic review aims to synthesise and critically evaluate recent evidence on advanced analytical technologies in elite sport, guided by three research questions:

- How are advanced analytical technologies distributed across application domains and sport disciplines, and where is technological maturity most evident?
- What ethical, regulatory, and operational barriers condition the sustainable adoption of these technologies in elite sport organisations?
- To what extent does increasing reliance on AI-based models affect human expert judgement in tactical and medical decision-making?

To structure this synthesis, the following section introduces a conceptual framework integrating technological capabilities, functional objectives, and contextual constraints, providing the analytical foundation for the Methods, Results, and Discussion sections that follow.

## Conceptual framework

Elite sport performance is increasingly shaped by the integration of physiological, cognitive, and technological dimensions. Traditional performance models have primarily focused on physical conditioning, load management, and injury prevention, relying on biomechanical, physiological, and epidemiological indicators (Kalén et al., 2019; Zouhal et al., 2021). While these approaches remain foundational, recent evidence highlights their limitations when applied in isolation, particularly in complex, high-performance environments where contextual, perceptual, and tactical factors interact dynamically (Yi et al., 2019).

Technological advancements have progressively expanded performance analysis beyond descriptive statistics toward continuous monitoring and data-driven decision-making. Wearable sensors and smart devices enable longitudinal tracking of physiological states, recovery, and readiness, supporting individualized performance management (Fiore et al., 2024; Medina-Ramírez et al., 2024). However, the increasing volume and heterogeneity of data necessitate advanced analytical frameworks capable of capturing non-linear relationships.

Machine learning (ML) methods have therefore emerged as a central pillar in contemporary performance and injury research. Predictive models applied to injury risk estimation and training adaptation demonstrate promising potential but remain constrained by class imbalance, limited generalizability, and challenges related to interpretability and real-world deployment (Dandrieux et al., 2023a; Dandrieux et al., 2023b; Tondut et al., 2023; Van Eetvelde et al., 2021). These limitations underline the need for cautious integration of ML outputs into applied decision-making.

In parallel, immersive technologies such as virtual reality (VR) have gained attention for their ability to provide representative training and assessment environments. Empirical studies indicate that VR can replicate perceptual–cognitive demands and support decision-making and skill acquisition, with acceptable levels of ecological validity (Kittel et al., 2019; van Biemen et al., 2023; Pastel et al., 2023). VR thus complements data-driven analytics by targeting cognitive and motor learning processes that are difficult to capture through sensor data alone.

More recently, generative artificial intelligence (GenAI) has introduced a new layer of abstraction, enabling tactical visualization, automated training design, and personalized guidance. While early evidence suggests pedagogical and operational benefits (Liu, 2024), systematic assessments caution against over-reliance on generative outputs due to risks related to hallucinations, lack of contextual specificity, and insufficient validation (Puce et al., 2025).

Taken together, this framework conceptualizes elite sport performance as emerging from the interaction between physiological monitoring, advanced analytics (ML), immersive environments (VR), and generative AI systems. By explicitly linking technological capabilities to functional objectives and contextual constraints, this integrative perspective provides the analytical lens for the present systematic review. It informs the selection of studies, the classification of technologies and application domains, and the interpretation of findings across performance, injury-related, and emerging objectives. The following Methods section details how this framework guided the search strategy, study selection, data extraction, and synthesis procedures.

## Methods

### Study design

This study is a systematic review synthesizing technology-enabled approaches for performance enhancement and injury-related outcomes in elite sport. The review examined three methodological pillars: (i) advanced statistical analysis, (ii) artificial intelligence (AI), including machine learning (ML), deep learning (DL), and generative AI (GenAI), and (iii) virtual and augmented reality (VR/AR). Two outcome domains were considered: performance improvement and injury prevention, management, and rehabilitation.

A systematic review methodology was adopted to ensure transparent, reproducible, and comprehensive identification and synthesis of relevant evidence. Owing to substantial heterogeneity in study designs, sports contexts, datasets, and outcome metrics, quantitative meta-analysis was not feasible; therefore, a structured qualitative synthesis was performed.

In this review, elite sport refers to organized competitive sport at the professional, semi-professional, or university level. High-performance stakeholders were eligible when directly involved in performance optimization or injury-related processes (e.g., athletes, coaches, referees, technical or medical support staff).

### Protocol registration

Protocol registration was not undertaken due to the exploratory scope of emerging technologies and the rapid evolution of the field during the review period.

### Information sources

A systematic and comprehensive literature search was conducted across four electronic databases: Google Scholar, Scopus, Web of Science, and IEEE Xplore. The search covered studies published between January 2019 and March 2025. Eligible publications were limited to English, French, and Spanish. Full-text articles were accessed through open-access repositories or institutional library subscriptions; no articles were obtained through paid access. The detailed database coverage and search dates are reported in Table 1.

**Table 1:** An overview of the search strategy adopted in the present systematic review.

Databases	Google Scholar, Scopus, Web of Science, and IEEE Xplore
Search string	(“statistical analysis” OR “machine learning” OR “deep learning” OR “artificial intelligence” OR “generative AI” OR “virtual reality” OR “immersive training” OR “augmented reality”). AND (“performance” OR “injury”) AND (“sport” OR “athlete”).
Inclusion Criteria	- Studies focusing on elite, high-performance, or competitive sport - Target populations including professional, semi-professional, or university-level athletes, as well as coaches, referees, or technical staff when sports performance is directly involved - Studies aiming to enhance sports performance, including physical, technical, tactical, cognitive, or decision-making aspects - Studies addressing the prevention, prediction, management, or rehabilitation of sports-related injuries - Use of at least one of the following approaches: advanced statistical analyses machine learning (ML) deep learning (DL) generative artificial intelligence (GenAI) virtual reality (VR) or augmented reality (AR) - Empirical studies employing quantitative or qualitative methods (experimental or observational designs) - Systematic reviews or narrative literature reviews providing a relevant methodological or conceptual contribution to the field
Exclusion criteria	- Studies involving non-sporting populations or exclusively recreational physical activity - Educational or school-based contexts without a competitive performance objective - Studies addressing neither sports performance nor injury prevention or management - Research focusing exclusively on sports marketing, media, economics, or sports law - Studies lacking advanced statistical analyses, AI / ML / DL / GenAI, or VR / AR components - Purely descriptive technological reports without application, validation, or scientific analysis - Non-scientific or non-academic documents, including opinion pieces or popular science articles
Language filter	English, French, Español
Time filter	Jan 2019 to Mar 2025

### Search strategy

The search strategy was defined *a priori* and implemented using Boolean logic to combine technology-related terms, immersion-related terms, and sport-related outcome terms. The core search string was:

(“statistical analysis” OR “machine learning” OR “deep learning” OR “artificial intelligence” OR “generative AI” OR “virtual reality” OR “immersive training” OR “augmented reality”)

AND (“performance” OR “injury”)

AND (“sport” OR “athlete”). Database-specific syntax and filters were adapted for each platform and are detailed in Table 1. In addition, backward citation tracking was performed by screening reference lists of key included studies, and forward citation tracking was conducted using citation indexes. All records identified through citation tracking were screened using the same predefined eligibility criteria as database records.

Additional articles were identified through backward and forward citation tracking of key publications.

### Eligibility criteria

Eligibility criteria are summarized in Table 1. Briefly, we included studies that: (1) addressed elite/high-performance/competitive sport; (2) targeted performance enhancement (physical, technical, tactical, cognitive, or decision-making) or injury prevention/management/rehabilitation; and (3) used at least one of the following approaches: advanced statistical analyses, ML/DL/GenAI, VR/AR.

We included empirical studies (quantitative or qualitative, experimental or observational) and review papers providing methodological or conceptual contributions. We restricted inclusion to peer-reviewed publications.

We excluded studies focused on non-sporting populations or exclusively recreational activity, school-based contexts without competitive objectives, papers not addressing performance or injury outcomes, work focused exclusively on marketing/media/economics/law, studies without advanced statistical/AI/VR components, purely descriptive technological reports without validation, and non-academic documents (Table 1).

### Study selection process

**Figure 1:**
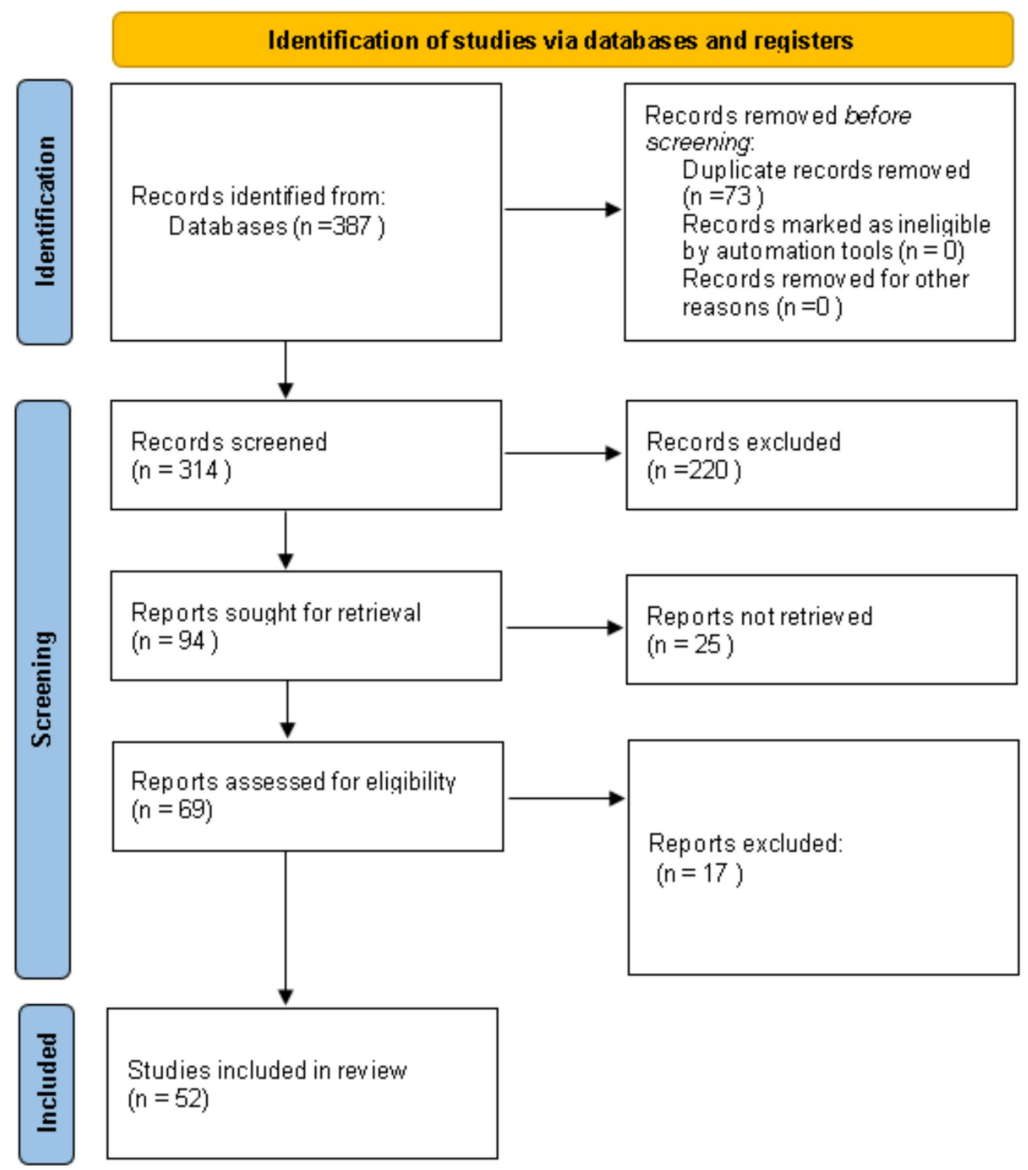
PRISMA 2020 flow diagram for the study selection process of the systematic review, including database and register searches.

Titles and abstracts were screened for relevance. Full texts were then assessed for eligibility. The study selection is reported using a PRISMA 2020 flow diagram (Figure 1).

Database searching identified 387 records. After removing 73 duplicates, 314 records were screened. We excluded 220 records at title/abstract stage. We sought retrieval of 94 full-text reports. A total of 25 reports were not retrieved due to access constraints (not available through open access or institutional access). We assessed 69 full-text reports for eligibility. We excluded 17 full-text reports. The final corpus comprised 52 studies.

### Screening transparency

Screening was performed by one reviewer using predefined criteria. A second author verified consistency on a subset of records.

### Data extraction and analysis

Data extraction followed a standardized grid. We extracted: sport discipline and sport type (team/individual/mixed), sample characteristics, dataset source and size, study objective, methodological family, analytical method/model, and key reported metrics and outcomes (Table 2). For empirical studies, we recorded model architectures and evaluation metrics when reported. For review and conceptual papers, we extracted the proposed frameworks and the main methodological contributions.

**Table 2:** Critical appraisal of included studies using a six-criterion checklist (C1–C6; 0–2 per criterion; total score 0–12).

**Ref**	**C1_Objectifs (0-2)**	**C2_Echantillon (0-2)**	**C3_Description Technique (0-2)**	**C4_Mesures Metriques (0-2)**	**C5_Biais Validite (0-2)**	**C6_Conclusions (0-2)**	**Score Total (/12)**	**Quality level**
Dandrieux et al. (2023b), Tondut et al. (2023), Fiore et al. (2024), and Mascret et al. (2022)	2	2	2	2	2	2	12	High
Kalén et al. (2019), Postupaiev et al. (2024), Zouhal et al. (2021), Wang et al. (2024), Tan et al. (2025) and Takamidoa et al. (2025)	2	2	2	2	1	2	11	High
Baladaniya and Choudhary (2025)	2	2	1	2	2	2	11	High
Rahimian and Toka (2022)	2	2	2	1	2	2	11	High
Van Eetvelde et al. (2021)	2	1	2	2	2	2	11	High
Ayodeji and Abiodun (2024), Peña-González et al. (2024), Kittel et al. (2019), Cui and Wang (2025), Chandra et al. (2024), Al-Majnoni et al. (2025), Ju et al. (2023), Keskar et al. (2019), Li and Zhang (2021), Chen (2025) and Baughman et al. (2025)	2	1	2	2	1	2	10	High
Moustakas (2025), Puce et al. (2025) and Westerbeek and Van Schaik (2025)	2	2	1	1	2	2	10	High
van Biemen et al. (2023) and Nambi et al. (2020)	2	1	2	2	1	2	10	High
Dandrieux et al. (2023a), Kadhim et al. (2025), and Liu (2024)	2	1	1	2	1	2	9	High
Sun (2022) and Naik et al. (2022)	2	1	2	1	1	2	9	High
Xu and Tang (2021)	2	0	2	2	1	2	9	High
Rusmanto et al. (2023)	2	1	1	2	1	1	8	Moderate
Branquinho et al. (2023) and Demeco et al. (2024)	2	1	1	1	1	2	8	Moderate
Medina-Ramírez et al. (2024)	2	0	2	1	1	1	7	Moderate
Tooth et al. (2024)	2	0	1	1	1	2	7	Moderate
Westerbeek (2025)	2	0	1	0	2	2	7	Moderate
Zhao and Xueying Guo (2022)	2	0	2	0	1	1	6	Moderate
Lian (2024)	1	1	1	0	0	1	4	Low

### Quality appraisal and risk of bias

A structured quality appraisal was conducted to contextualize the strength of evidence across the included studies. Each study had been previously assessed using a six-criterion grid scored from 0 to 2 per criterion, yielding a total quality score ranging from 0 to 12 (Table 3). The criteria covered: clarity of objectives (C1), sample description (C2), technical description (C3), measures and metrics (C4), bias and validity considerations (C5), and conclusions (C6).

**Table 3:** Summary of the included literature (N = 52), classifying studies by methodology, research objective, and dataset characteristics, (ML = Machine learning, DL = Deep Learning, SL = Statistical modelling, GAI = Generative AI, HY = Hybrid (ML + DL OR DL + GAI), VR = Virtual Reality, Ind = Individual, Ath/Ply = athletic/player, MOTA = Multiple Object Tracking Accuracy, mAP = mean Average Precision, N/A = Not Available).

**Ref**	**DOI**	References	**Objective**	**Methodological family**	**Analytical method / Model**	**Sport type**	**Sport discipline**	**Data source**	**size**	**Type**	**Key reported metrics**
1.	https://doi.org/10.1016/j.jts.2023.04.004	Dandrieux et al. (2023a)	Perceptions and beliefs	SL	Analysis and synthesis of bibliographic data	Team	Football	2022 European Championships	79	Survey	Interest: 85±16, Stress: 41±33
2.	https://doi.org/10.18060/27691	Ayodeji and Abiodun (2024)	Rivalry-performance	SL	MANCOVA	Team	Football	messivsronaldo.net	19	Saisons	Positive effect on performance
3.	https://doi.org/10.1016/j.jts.2023.04.003	Dandrieux et al. (2023b)	Injuries	ML	Standardized injury reporting form	Team	Football	professional or semi-professional teams	284	Ath/Ply	F1-score: 0.33
4.	https://doi.org/10.1016/j.jts.2023.04.002	Tondut et al. (2023)	Injuries	ML	Self-reporting via Athlete360 app	Mixed	Athletes	data collected from athletes	3688	Observations	Accuracy: 91%
5.	https://doi.org/10.1186/s40634-021-00346-x	Van Eetvelde et al. (2021)	Injuries	ML	Systematic Review	Mixed	Athletes	Academic Databases	11	studies	Varied methods and performance
6.	https://doi.org/10.3389/fpsyg.2019.00076	Kalén et al. (2019)	aging	SL	ANOVA	Team	Football	UEFA and Transfermarkt	16062	Ath/Ply	Average age: 24.9 to 26.5 years old
7.	https://doi.org/10.3389/fpsyg.2019.02738	Yi et al. (2019)	Performance	SL	Observations	Team	Football	whoscored.com	9799	Observations	Minimal differences between leagues
8.	https://www.mdpi.com/2076-3417/14/23/10778	Fiore et al. (2024)	Performance	SL	Meta-Analysis	General	General	Academic Databases	19	studies	HR bias: -0.4 bpm
9.	https://www.mdpi.com/2673-7140/4/2/14	Medina-Ramírez et al. (2024)	Sports recovery	SL	Statistical analysis (software: SPSS)	Team	Basketball	Oura ring Data and biochemical tests	12	Ath/Ply	Improved sleep (p<0.001)
10.	https://doi.org/10.3390/ai5020042	Postupaiev et al. (2024)	Improve dissemination	DL	YOLOv8	Team	Football	Various online sources	7 500	Media	Accuracy: 97.78%
11.	https://doi.org/10.1155/2022/1339434	Zhao and Xueying Guo (2022)	VR training	VR	Qualitative analysis of VR intervention	Team	Football	N/A	N/A	N/A	Potential for improvement
12.	https://doi.org/10.33689/spormetre.1249071	Karabıyık and Tugay (2023)	Collective behaviors	SL	ANOVA	Team	Football	FIFA 2022	64	Matches	Importance of scoring goals
13.	https://doi.org/10.3390/sports11090174	Branquinho et al. (2023)	Weather impact	SL	Descriptive and inferential statistics	Team	Football	Academic Databases	52	studies	No significant negative effect
14.	https://doi.org/10.1016/j.scispo.2021.03.001	Zouhal et al. (2021)	Injuries	SL	Narrative literature review	Team	Football	Academic Databases	205	studies	Need for individualized strategies
15.	https://doi.org/10.1016/j.scispo.2023.10.003	Peña-González et al. (2024)	Age-performance analysis	SL	ANOVA	Team	Football	Spanish national competition	75	Ath/Ply	No systematic difference
16.	https://doi.org/10.1016/j.jts.2024.06.002	Tooth et al. (2024)	Injuries	SL	Bibliographic research	Mixed	General	Concussions in the United States	1000	Ath/Ply	Essential prevention
17.	https://doi.org/10.3390/app132312965	Cossich et al. (2023)	Sports technologies	VR	Narrative literature review	Mixed	Athletes	Narrative review	N/A	N/A	Performance optimization
18.	https://doi.org/10.3390/socsci14030174	Moustakas (2025)	Sport for Development	GAI	Thematic Analysis	General	General	Survey	43	Survey	Capacity building vs. "Generic" programming risks
19.	https://doi.org/10.1016/j.psychsport.2022.102201	Mascret et al. (2022)	Acceptance VR athletes	SL	Questionnaire + CFA + ANOVA	Mixed	Athletes	Survey	1162	Ath/Ply	High intention to use
20.	https://doi.org/10.1016/j.jsams.2019.03.012	Kittel et al. (2019)	VR assessment-decision	VR	Validation study	Team	Australian football	Video clips	120	Media	Accuracy: 75.18%
21.	https://doi.org/10.1016/j.humov.2023.103091	van Biemen et al. (2023)	Refereeing representation	VR	comparative study	Team	Football	Data collected	15	Ath/Ply	Accurate behavior in VR
22.	https://doi.org/10.47197/retos.v50.100319	Rusmanto et al. (2023)	Sports commitment	VR	Mann-Whitney U test	Team	Football	Junior male athletes	40	Ath/Ply	Significant improvement
23.	https://doi.org/10.1155/2020/2981273	Nambi et al. (2020)	Chronic pain	VR	Comparative study	Team	Football	Male college players	45	Ath/Ply	Pain reduction
24.	https://doi.org/10.1007/s10055-022-00679-7	Pastel et al. (2023)	Learning	VR	ANOVA	Ind	Karate	Custom-collected data	83	Ath/Ply	Effectiveness comparable to video
25.	https://doi.org/10.3390/medicina60061000	Demeco et al. (2024)	injuries	VR	Narrative literature review	Team	Football	PubMed, Scopus, Web of Science	__		Potential for rehabilitation
26.	https://doi.org/10.1371/journal.pone.0317414	Cui and Wang (2025)	Pattern recognition (sprint)	ML	Decision tree with cross-validation	Ind	Athletes	Simulated sprint data	100	Media	Accuracy: 94.9%, Recall: 0.91, F1 score: 0.93
27.	https://doi.org/10.1038/s41467-024-45965-x	Wang et al. (2024)	Tactical assistance	HY	TacticAI model (GNN)	Team	Football	Premier League (2020-2021)	7176	Media	Accuracy: 78.2%, F1-score: 0.71, Expert preference: 90%
28.	https://doi.org/10.1155/2022/1205622	Sun (2022)	College athletic performance	HY	Prediction framework with ML classification	Mixed	Athletes	Chinese university	100	Ath/Ply	Accuracy: 91.7%
29.	https://doi.org/10.21203/rs.3.rs-3995768/v1	Chandra et al. (2024)	Performance prediction	ML	ML classification based on historical data	Team	Football	Public Databases / APIs	N/A	Ath/Ply	Accuracy: 65%
30.	https://doi.org/10.48084/etasr.9309	Al-Majnoni et al. (2025)	Performance	DL	Object detection (YOLO) on videos	Ind	Swimming	Public dataset + collected data	2720	Media	mAP (style): 0.98, mAP (style+defects): 0.95
31.	https://doi.org/10.1515/jqas-2020-0088	Rahimian and Toka (2022)	Optical tracking	DL	Narrative literature review	Team	Football	Academic Databases	50	studies	Qualitative comparison of methods
32.	https://doi.org/10.3390/app132011227	Ju et al. (2023)	Performance	HY	Pose refinement network	Ind	Golf	Public Dataset (3DPW) + collected data	40	Media	MPJPE reduction: ~9%
33.	https://doi.org/10.4108/eetpht.10.5809	Lian (2024)	Performance	DL	Performance prediction using an ML model	Ind	Athletes	Academic data	270	Ath/Ply	Accuracy: 92.6%, Recall: 94.3%
34.	https://doi.org/10.1109/ACCESS.2022.3161441	Naik et al. (2022)	Tracking players and referees	HY	Object detection and tracking in videos	Team	Football	ISSIA et SoccerNet	10,217	Media	MOTA: 96%
35.	https://doi.org/10.55860/PUOR4953	Kadhim et al. (2025)	Technical evaluation	HY	Extraction of motor parameters via CNN	Ind	Karate	Athlete videos	50	Media	Accuracy: 89%-95%
36.	https://doi.org/10.1016/j.neucom.2022.01.098	Wang et al. (2022)	Gameplay prediction	ML	Descriptive analysis + Supervised prediction	Team	Basketball	NBA open-source data	39	Saisons	Effectiveness of explainable AI in predicting results with interpretation
37.	https://doi.org/10.3390/s25072260	Wesely et al. (2025)	Performance	HY	Gaussian Process Classification	Team	Cheerleading	Sensors (Wearables / IMU)	1,102	Media	Accuracy: 90%
38.	https://doi.org/10.23950/jcmk/16412	Baladaniya and Choudhary (2025)	Performance and Injury Prevention	HY	Systematic Review	Mixed	General	Academic Databases	36	studies	AI is an integral tool, future focus on validation and ethics
39.	https://doi.org/10.1016/j.opelre.2019.02.003	Keskar et al. (2019)	Object tracking (ball)	DL	Video detection and tracking	Team	Football	Public Dataset ISSIA	6	Media	Position Error < 20 pixel
40.	https://doi.org/10.1155/2021/4485589	Li and Zhang (2021)	Performance	HY	Evaluation of a new NN algorithm	Team	Basketball	Custom image collection	1000	Media	Accuracy: >90%
41.	https://doi.org/10.3389/fnbot.2020.620378	Xu and Tang (2021)	injuries	ML	Improved Q-Learning with Fuzzy Controller	Team	Basketball	Simulation / Sensors	N/A	Simulation	Robot navigates faster
42.	https://doi.org/10.1080/23750472.2024.2449016	Jud and Thalmann (2024)	Performance	HY	Systematic Review	Mixed	General	Academic Databases	40	studies	High potential, need for pedagogical validation
43.	https://doi.org/10.3390/app15105672	Guneralp et al., 2025	Performance	ML	Experimental study with pre/post-tests	Team	Football	Custom-collected data	60	Ath/Ply	Accuracy (XGBoost): 100% for distinguishing between groups
44.	https://doi.org/10.1142/S0129156425500016	Chen (2025)	Performance	HY	Proposal for an ML architecture (AGTO-SELM)	Mixed	General	Kaggle ("IoT-driven-sports")	2500	Data	Accuracy: 97.4%
45	https://doi.org/10.1371/journal.pone.0318321	Tan and Chen (2025)	Generating training plans	GAI	GANs	Ind	General	Olympic Sports Dataset	1000	Media	MSE reduction: 22% / Generation Time: -45%
46	https://doi.org/10.3390/app15073497	Puce et al. (2025)	Exercise Prescription	GAI	Systematic Review	Ind	General	UnoPerTutto Database	10	studies	Highlighted risks of hallucinations in medical/training advice
47	https://doi.org/10.3389/fspor.2025.1597444	Westerbeek (2025)	Fan Engagement	GAI	Narrative Literature Review	General	General	Academic Databases	N/A	N/A	Risks Identified: Erosion of autonomy, Algorithmic bias, Gambling triggers
48	https://doi.org/10.3389/fspor.2025.1642180	Westerbeek and Van Schaik (2025)	Branding & Governance	GAI	Systematic Review	General	General	Academic Databases	47	studies	Power asymmetry (Platforms vs Athletes); Need for decentralized data rights
49	https://doi.org/10.1145/3637528.3671542	Baughman et al. (2024)	Automated Commentary	GAI	LLM + RAG	Mixed	Golf, Tennis, Football	US Open, Wimbledon, Masters, ESPN	3363	Media	Rouge-L: 82.00; Speed improvement: 15x; Perplexity: 6.6
50	https://doi.org/10.2478/amns-2024-1915	Liu (2024)	Tactical Education	GAI	Generative Big Model + ANOVA	Team	Basket-ball	College Students	60	Ath/Ply	Improvement: +4.3 pts in tactical awareness (p<0.05)
51	https://doi.org/10.48550/arXiv.2510.10496	Takamidoa et al. (2025)	Personalized Motion Guidance	GAI	Transformer-VAE	Team	Baseball	lancer de baseball collectés	255	throws	RMSE: 3.2 cm; Smooth Style Transfer (DTW verified)
52	https://doi.org/10.48550/arXiv.2505.09024	Baughman et al. (2025)	Human-AI Alignment	GAI	LLM-as-a-Judge + Meta-Prompting	Individual	Tennis	US Open 2024	254	Matches	Alignment: 53.8% (100% match with human intent); Iterations: 4.38 avg

For synthesis purposes, studies sharing identical quality score profiles were grouped together in the summary table to improve readability and transparency. No modification or re-weighting of the original scores was performed.

Quality scores were used solely to interpret the strength and robustness of the evidence and were not applied as exclusion criteria. Based on the total score, studies were classified into three quality levels: high quality (9–12 points), moderate quality (6–8 points), and low quality (0–5 points). This categorization was used to support qualitative interpretation of findings and to contextualize potential risks of bias across studies.

### Synthesis approach

Heterogeneity in designs, sports, datasets, and outcome metrics prevented quantitative meta-analysis. We therefore performed a thematic synthesis. Studies were grouped by primary technology pillar (statistical analysis, ML/DL/GenAI, VR/AR, hybrid). Objectives were coded as performance-related, injury-related, or other. Findings were integrated across pillars to identify recurring applications, methodological patterns, and gaps. Quality appraisal results were used to contextualize the strength of evidence during interpretation.

### Methodological limitations

This systematic review was conducted within predefined temporal, database, and language boundaries, which may have resulted in the omission of relevant studies published outside these parameters. The included studies comprised heterogeneous designs, sports contexts, datasets, and outcome measures, limiting direct quantitative comparability and precluding meta-analysis. Although a structured screening and quality appraisal process was applied, initial title and abstract screening was conducted by a single reviewer, which may have introduced selection bias despite secondary verification procedures.

## Results

### Study selection and characteristics

After application of the eligibility criteria and screening process described in the Methods section, 52 studies were included in the final synthesis. These studies encompass a wide range of research designs, sports contexts, and technological approaches applied to performance enhancement and injury-related outcomes in elite sport.

The main characteristics of the included studies are summarised in Table 2, which reports, for each article, the methodological family, research objective, sport type, sport discipline, data source, sample size, study type, and key reported metrics. The included studies, randomized controlled trials, observational studies, and systematic or narrative reviews, reflecting the methodological diversity of the field.

Team sports were predominant, particularly football, followed by basketball, athletics, combat sports, swimming, baseball, and mixed-sport contexts. Sample sizes varied substantially, ranging from small experimental cohorts (e.g., intervention studies with fewer than 20 participants) to large-scale datasets involving thousands of athletes, matches, or media samples (Kalén et al., 2019; Wang et al., 2024). Several studies relied on secondary or public databases, whereas others used custom-collected sensor, video, or survey data (Medina-Ramírez et al., 2024; Guneralp et al., 2025).

Research objectives were primarily oriented toward performance improvement and injury prevention, prediction, or rehabilitation, with a smaller subset addressing related themes such as tactical analysis, technology acceptance, or governance issues.

### Distribution of technological approaches

**Figure 2:**
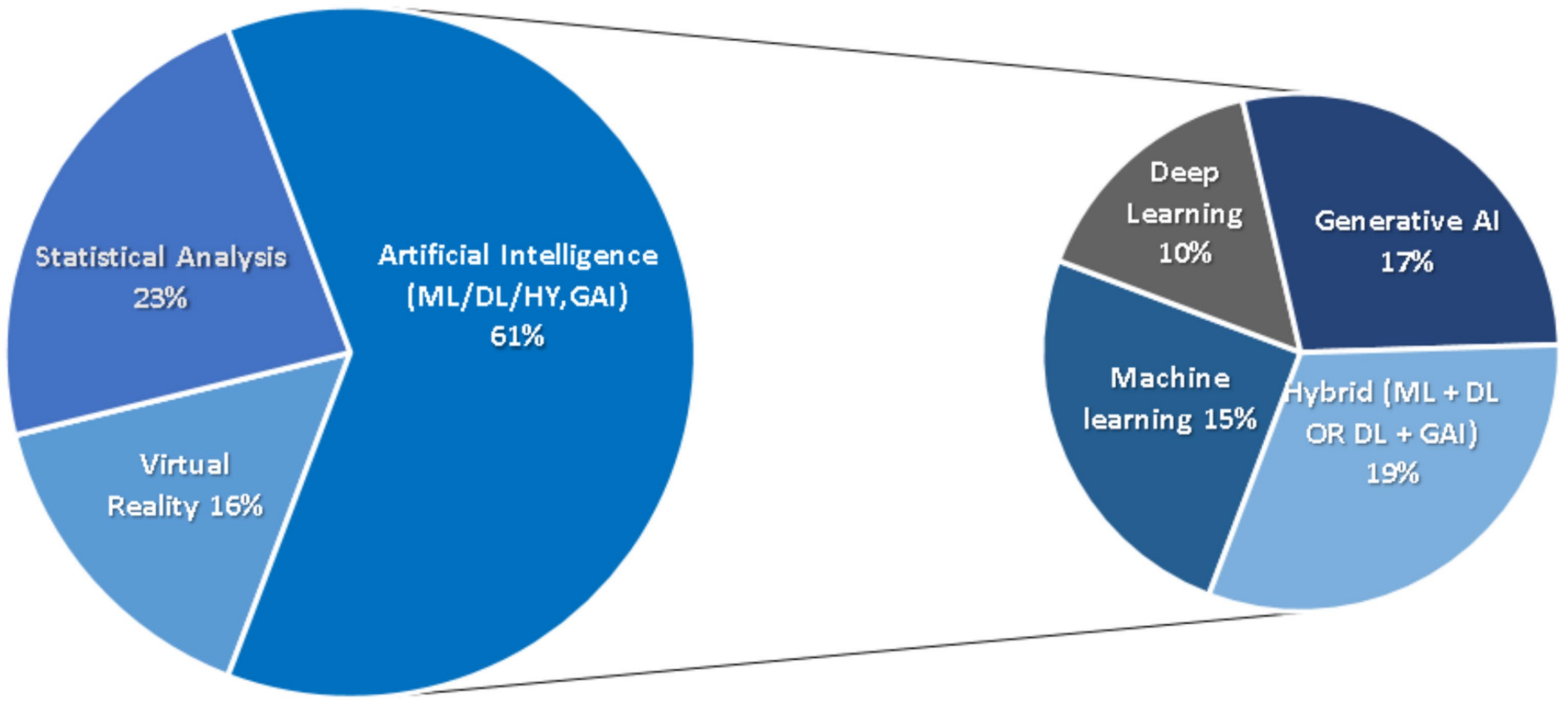
2D pie of pie chart displaying the distribution of selected studies by primary technology approach.

As shown in Figure 2, the distribution of studies according to their primary technological approach indicates a clear predominance of Artificial Intelligence–based methods. These approaches, encompassing machine learning (ML), deep learning (DL), generative AI (GAI), and hybrid frameworks, represent the largest proportion of the included studies, followed by statistical analysis and virtual reality (VR).

Statistical modelling approaches were used in 12 studies, typically to test hypotheses related to performance determinants, aging effects, or collective behaviors (Ayodeji and Abiodun, 2024; Kalén et al., 2019; Karabıyık and Tugay, 2023). Virtual reality interventions were reported in 8 studies, focusing on training, assessment, rehabilitation, or user acceptance (Mascret et al., 2022; Kittel et al., 2019; Rusmanto et al., 2023).

Machine learning approaches accounted for 8 studies, while deep learning techniques were applied in 5 studies, mainly for video-based tracking, technical assessment, or prediction tasks (Al-Majnoni et al., 2025; Naik et al., 2022). Generative AI was explicitly addressed in 9 studies, covering training plan generation, content automation, tactical visualization, and human–AI interaction (Tan and Chen, 2025; Puce et al., 2025; Baughman et al., 2024). In addition, 10 studies adopted hybrid frameworks, combining ML, DL, or GAI components to leverage complementary strengths (Wang et al., 2024; Guneralp et al., 2025).

### Research objectives across sport contexts

**Figure 3:**
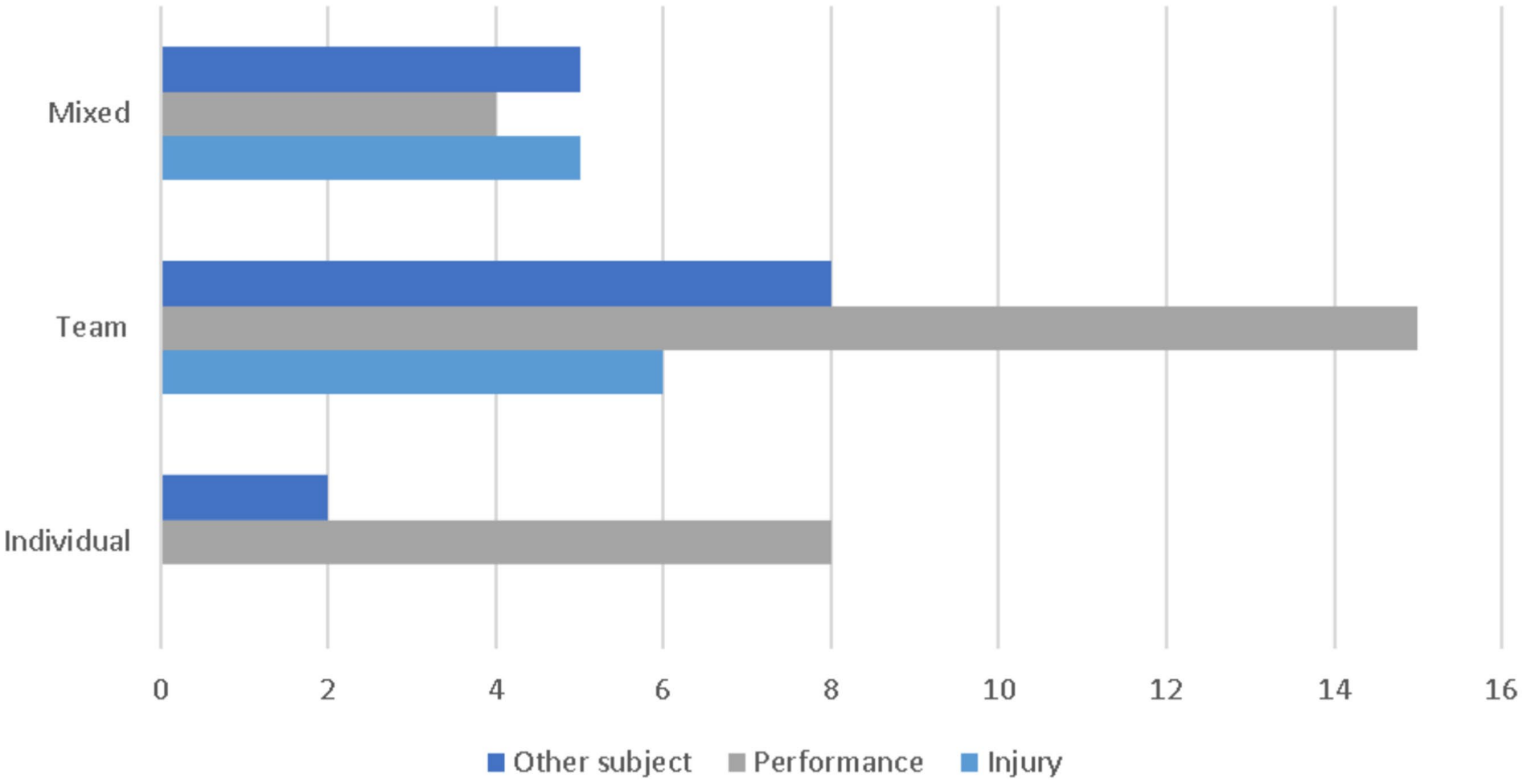
Grouped bar chart displaying the distribution of research objectives across sport types.

Figure 3 illustrates structural differences in research priorities between team-based and individual sport contexts. Research objectives were classified into three categories: performance enhancement, injury-related outcomes, and other objectives, the latter referring to studies that did not directly target performance improvement or injury prevention/management but instead addressed tactical analysis, technology acceptance, usability, governance, decision support, or methodological and conceptual developments.

Across the entire corpus, performance enhancement was the most frequently investigated objective, regardless of sport context.

In team sports, performance-oriented studies were predominant (15 studies), alongside a substantial number addressing injury-related outcomes (6 studies). A notable proportion of team-based research focused on other objectives (8 studies), including tactical and strategic analysis, fan or athlete engagement, and governance-related or decision-support applications. These studies frequently integrated physical, technical, and contextual data sources to capture the collective nature of team performance (Yi et al., 2019; Wang et al., 2024).

In individual sports, studies focused almost exclusively on performance enhancement (8 studies), with no injury-focused investigations identified in this subgroup. These studies typically examined technical execution, movement quality, or skill acquisition using video-based, sensor-based, or biomechanical data (Pastel et al., 2023; Ju et al., 2023).

Studies conducted in mixed-sport contexts showed a more balanced distribution across objectives, including injury-related outcomes (5 studies), performance enhancement (4 studies), and other objectives (5 studies). These investigations often adopted broader methodological, technological, or conceptual perspectives, such as cross-sport injury prediction frameworks or general evaluations of machine learning applicability in sport science (Van Eetvelde et al., 2021; Baladaniya and Choudhary, 2025).

### Alignment between technologies and research objectives

Statistical analysis was mainly applied to performance-related outcomes (7 studies) and injury-related questions (3 studies), with limited use for objectives outside these two core domains. This confirms its role as a hypothesis-driven and confirmatory approach rather than an exploratory one.

Virtual reality was employed for performance enhancement (4 studies) and injury management or rehabilitation (2 studies), but also for other objectives, defined here as studies primarily addressing technology acceptance, usability, ecological validity, or experiential dimensions rather than direct performance or injury outcomes. These included investigations of athlete acceptance of VR systems (Mascret et al., 2022) and narrative or conceptual analyses of VR-based rehabilitation environments (Demeco et al., 2024).

Machine learning approaches were most frequently associated with performance prediction and training analysis (9 studies), but also with injury prediction (4 studies) and a smaller number of secondary objectives (2 studies), such as methodological validation or feasibility assessments (Dandrieux et al., 2023a; Dandrieux et al., 2023b; Guneralp et al., 2025).

Deep learning was predominantly applied to performance-related tasks (5 studies), with fewer injury-focused applications (1 study) and some exploratory uses (2 studies) involving automated tracking, detection, or broadcast-oriented tasks rather than athlete-centered outcomes (Naik et al., 2022; Keskar et al., 2019).

Generative AI showed a distinct objective profile, with a relatively balanced distribution between performance-related applications (4 studies) and other objectives (5 studies). In this category, other objectives mainly referred to content generation, training-plan synthesis, decision-support tools, methodological validation, and human–AI alignment, rather than direct injury prevention or rehabilitation applications. Notably, no injury-focused studies were identified for generative AI within the included corpus (Puce et al., 2025; Baughman et al., 2024).

## Discussion

### Principal findings and contribution of this review

This systematic review synthesised evidence from 52 studies published between 2019 and 2025 examining technology-enabled approaches for performance enhancement and injury-related outcomes in elite sport. The results highlight a clear predominance of data-driven and computational approaches, with artificial intelligence (AI)–based methods, including machine learning (ML), deep learning (DL), and generative AI (GAI), representing the largest share of the literature. Statistical analysis and virtual reality (VR) remain important complementary pillars, addressing more established analytical questions and immersive training or rehabilitation contexts, respectively.

Across technologies and sport contexts, performance enhancement emerged as the dominant research objective, whereas injury-related applications, although present, were less consistently investigated. A third category of studies addressing other objectives, such as tactical analysis, technology acceptance, decision support, and methodological or conceptual development, reflects a diversification of research aims beyond direct performance or injury outcomes. This diversification is particularly evident in DL and GAI studies, suggesting a shift toward exploratory and system-level applications.

By integrating methodological characteristics, research objectives, and sport contexts, this review provides a structured overview of how emerging technologies are currently applied in elite sport and identifies patterns that were not systematically synthesised in previous reviews.

### Technology-specific trends and comparative interpretation

#### Artificial intelligence approaches

**Figure 4:**
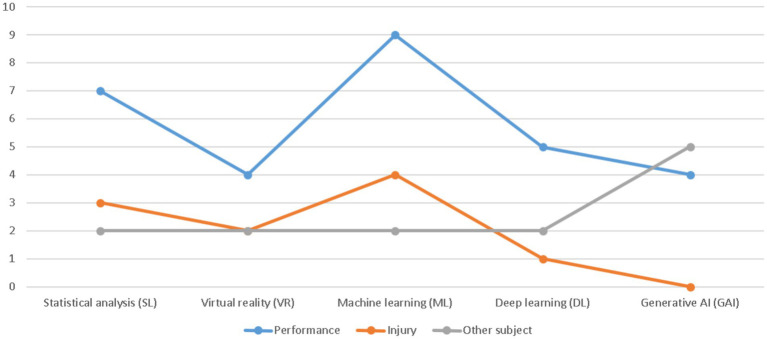
Illustrates the relationship between technological approaches and primary research objectives. Across all technological domains, performance improvement emerged as the dominant objective.

AI-based methods dominated the corpus, particularly for performance-related applications. ML models were most frequently used for performance prediction, training evaluation, and injury risk estimation. However, while several studies reported high global accuracy, detailed examination of performance metrics revealed substantial variability in precision, recall, and class imbalance handling (Dandrieux et al., 2023a; Dandrieux et al., 2023b; Tondut et al., 2023). This suggests that headline accuracy figures alone may overestimate practical utility, especially for injury prediction tasks where false negatives carry high clinical risk (Figure 4).

DL approaches were primarily applied to video-based and spatiotemporal data, enabling automated tracking, technical assessment, and tactical analysis (Naik et al., 2022; Keskar et al., 2019). Compared with ML, DL studies tended to focus less on predictive performance metrics and more on automation, objectivity, and scalability. However, the limited number of injury-focused DL applications indicates that these methods remain underexplored for health-related outcomes.

GAI studies showed a distinct profile, with applications centred on content generation, training synthesis, and human–AI alignment rather than direct injury or physiological outcomes (Puce et al., 2025; Baughman et al., 2024). This emerging trend highlights the potential of GAI to support decision-making, communication, and coaching workflows, while also raising questions about validation, transparency, and ethical deployment.

#### Statistical analysis and virtual reality

Statistical modelling remains foundational for hypothesis testing and explanatory analysis. In this review, statistical approaches were predominantly applied to performance and injury-related questions, with minimal use for exploratory or system-level objectives. This reflects the strength of statistical methods for interpreting structured datasets but also their limited capacity to address complex, high-dimensional data without integration with AI techniques.

VR studies demonstrated consistent benefits for skill acquisition, perceptual–cognitive training, and rehabilitation. Unlike AI-based approaches, VR research frequently employed controlled experimental designs, including randomised trials, providing stronger causal evidence for effectiveness (Rusmanto et al., 2023; Nambi et al., 2020). However, VR applications were constrained by cost, accessibility, and ecological validity concerns, limiting large-scale adoption in elite sport settings.

### Sport context and research objectives

The predominance of team sports reflects both data availability and the strategic value of technology for collective performance analysis. Team-based studies also exhibited greater thematic diversity, encompassing performance, injury management, and other objectives such as tactics and governance. In contrast, individual sport studies focused almost exclusively on performance enhancement, with a notable absence of injury-focused investigations. This gap suggests missed opportunities to apply predictive and preventive technologies in individual sport contexts, where injury risk can significantly impact career longevity.

Mixed-sport studies often adopted broader methodological perspectives, including systematic reviews and conceptual analyses, indicating their role in shaping cross-disciplinary understanding rather than delivering sport-specific interventions.

### Methodological quality, limitations, and research gaps

The quality appraisal revealed substantial heterogeneity in methodological rigor. High-quality studies were concentrated among controlled VR interventions and well-documented ML/DL applications with transparent datasets and evaluation metrics. Moderate- and low-quality studies frequently lacked clear sample descriptions, bias mitigation strategies, or validated outcome measures. These limitations restrict direct comparison across studies and partially explain the absence of quantitative meta-analysis.

Several critical gaps emerge from this synthesis. First, injury-related applications remain underdeveloped relative to performance-focused research, particularly for DL and GAI approaches. Second, few studies systematically address external validity, real-world deployment, or long-term effectiveness. Third, explainability and transparency are inconsistently incorporated, despite their importance for practitioner trust and ethical use.

### Ethical, regulatory, and governance considerations

Beyond methodological limitations, several ethical, regulatory, and governance challenges emerged across the reviewed studies and critically condition the sustainable adoption of advanced analytical technologies in elite sport. Algorithmic bias represents a central concern, as many AI and ML models are trained on sport-, gender-, or region-specific datasets, which may limit generalisability and amplify structural inequalities when deployed across diverse athlete populations (Van Eetvelde et al., 2021; Rahimian and Toka, 2022). This issue is particularly salient in injury prediction and performance modelling, where biased outputs may directly affect athlete health and career trajectories.

Data ownership, privacy, and informed consent also constitute major barriers, given the increasing reliance on wearable sensors, video tracking systems, and longitudinal athlete monitoring platforms. Several studies highlight unresolved questions regarding who controls athlete-generated data, how long such data are retained, and how compliance with evolving data protection regulations is ensured in high-performance environments (Fiore et al., 2024; Jud and Thalmann, 2024). These challenges are compounded when third-party technology providers are embedded within sport organisations.

Moreover, the integration of AI-based decision-support systems into tactical and medical workflows raises concerns related to accountability and over-reliance. When algorithmic recommendations conflict with expert judgement, responsibility for decision outcomes becomes ambiguous, particularly in high-stakes medical or return-to-play contexts (Dandrieux et al., 2023a; Dandrieux et al., 2023b). Recent work on generative AI further cautions against uncritical adoption due to risks of hallucination, lack of contextual grounding, and limited transparency of generative outputs (Puce et al., 2025; Baughman et al., 2024).

Collectively, these findings underscore the necessity of transparent, explainable, and ethically governed analytical systems that preserve human oversight and contextual expertise. Sustainable implementation of advanced technologies in elite sport therefore depends not only on technical performance but also on governance frameworks that balance innovation, athlete protection, and professional accountability.

### Implications for practice and future research

For practitioners, the findings suggest that AI-based tools offer promising support for performance analysis and training optimisation, but their outputs must be interpreted cautiously, especially in injury prediction contexts. VR appears well-suited for controlled training and rehabilitation environments but requires integration with sport-specific constraints to maximise ecological validity.

Future research should prioritise (i) rigorous validation of AI models using clinically and practically meaningful metrics, (ii) expansion of injury-focused applications across sport types, (iii) integration of explainable AI to enhance trust and adoption, and (iv) longitudinal studies assessing real-world effectiveness. Addressing these gaps will be essential for translating technological innovation into sustainable performance and health benefits in elite sport.

## Conclusion

This systematic review synthesised evidence from 52 studies published between 2019 and 2025 to examine technology-enabled approaches for performance enhancement and injury-related outcomes in elite sport. By integrating methodological characteristics, research objectives, and sport contexts, the review provides a structured overview of how statistical analysis, artificial intelligence, and virtual reality are currently mobilised across elite sport research.

The findings indicate a clear predominance of performance-oriented applications, particularly those based on machine learning and deep learning, while injury-related outcomes remain comparatively underrepresented and methodologically heterogeneous. Statistical analysis continues to play a central role in explanatory and hypothesis-driven research, whereas virtual reality demonstrates consistent effectiveness for skill acquisition, perceptual–cognitive training, and rehabilitation within controlled experimental settings. Generative AI emerges as a distinct and rapidly developing domain, primarily addressing decision support, content generation, and training design, but currently lacking validation for direct performance or health outcomes.

Despite the growing volume of research, this review highlights important limitations in the existing literature. These include substantial variability in study quality, inconsistent reporting of evaluation metrics, limited external validation, and a frequent reliance on accuracy-based indicators that may overestimate practical utility, particularly for injury prediction. Furthermore, the dominance of team sport contexts and performance-focused objectives suggests that several sport types and health-related applications remain insufficiently explored.

From a practical perspective, the evidence supports the cautious adoption of AI-based tools for performance analysis and training optimisation, alongside VR-based interventions for targeted training and rehabilitation. However, translation into applied elite sport settings requires greater emphasis on explainability, robustness, and real-world effectiveness. For researchers, future work should prioritise rigorous study designs, transparent quality assessment, clinically meaningful outcome measures, and longitudinal validation. Expanding injury-focused research, integrating explainable AI frameworks, and addressing ethical and governance considerations will be critical for sustainable implementation.

Overall, this review contributes a comprehensive and critically grounded synthesis of contemporary technological approaches in elite sport. By clarifying current trends, methodological strengths and weaknesses, and key research gaps, it provides a foundation for more robust, transparent, and impactful future research at the intersection of performance science, athlete health, and emerging technologies.

## Data Availability

The original contributions presented in the study are included in the article/supplementary material, further inquiries can be directed to the corresponding author.

## References

[ref1] Al-Majnoni A. Al-Sahli J. Al-Ahmady D. Al-Mutairi A. Alsini A. Alharbi M. (2025). Moar: a swimmer motion swimming style identification model using deep learning. Eng. Technol. Appl. Sci. Res. 15, 19295–19302. doi: 10.48084/etasr.9309

[ref2] Ayodeji I. Abiodun O. (2024). The effect of rivalry on sport performance: a case study of Cristiano Ronaldo and Lionel Messi. Sports Innov. J. 5, 61–77. doi: 10.18060/27691

[ref3] Baladaniya M. Choudhary A. K. (2025). Artificial intelligence in sports science: a systematic review on performance optimization, injury prevention, and rehabilitation. J. Clin. Med. Kazakhstan 22, 64–72. doi: 10.23950/jcmk/16412

[ref4] Baughman A. Agarwal R. Morales E. Akay G. (2025). Automated Meta Prompt Engineering for Alignment with the Theory of Mind. arXiv. doi: 10.48550/arXiv.2505.09024

[ref5] Baughman A. Morales E. Agarwal R. Akay G. Feris R. Johnson T. . (2024). “Large scale generative AI text applied to sports and music,” in Proceedings of the 30th ACM SIGKDD Conference on Knowledge Discovery and Data Mining (4784–4792). doi: 10.1145/3637528.3671542

[ref41] Biemen Tammie van Müller Daniel Mann David L. Virtual reality as a representative training environment for football referees Hum. Mov. Sci. 89 (2023):103091 doi: 10.1016/j.humov.2023.103091 37084551

[ref6] Branquinho L. Forte P. Thomatieli-Santos R. V. de França E. Marinho D. A. Teixeira J. E. . (2023). Perspectives on player performance during FIFA world cup Qatar 2022: a brief report. Sports 11:174. doi: 10.3390/sports11090174, 37755851 PMC10534916

[ref9008] Chandra B. Jennet S. D. Keshav A. M. (2024). Prediction of football player performance using machine learning algorithm. Res. Sq. doi: 10.21203/rs.3.rs-3995768/v1

[ref7] Chen L. (2025). Application of internet of things data mining in sports teaching and training system optimization. Int. J. High Speed Electron. Syst. 35:2550001. doi: 10.1142/S0129156425500016

[ref8] Cossich V. R. A. Carlgren D. Holash R. J. Katz L. (2023). Technological breakthroughs in sport: current practice and future potential of artificial intelligence, virtual reality, augmented reality, and modern data visualization in performance analysis. Appl. Sci. 13:12965. doi: 10.3390/app132312965

[ref9] Cui G. Wang C. (2025). The machine learning algorithm based on decision tree optimization for pattern recognition in track and field sports. PLoS One 20:e0317414. doi: 10.1371/journal.pone.0317414, 39946363 PMC11824974

[ref10] Dandrieux P.-E. Navarro L. Chapon J. Tondut J. Hollander K. Edouard P. (2023a). Perceptions et croyances sur la prédiction des blessures en sports en tant que mesure de réduction des risques de blessure: une enquête en ligne sur les acteurs du sport de haut niveau (athlètes, entraîneurs, professionnels de santé). J. Traumatol. Sport. 40, 81–87. doi: 10.1016/j.jts.2023.04.004

[ref11] Dandrieux P. Tondut J. Nagahara R. Mendiguchia J. Morin J. Lahti J. . (2023b). Prédiction des blessures des ischiojambiers en football à l’aide d’apprentissage automatique: étude préliminaire sur 284 footballeurs. J. Traumatol. Sport. 40, 69–73. doi: 10.1016/j.jts.2023.04.003

[ref12] Demeco A. Salerno A. Gusai M. Vignali B. Gramigna V. Palumbo A. . (2024). The role of virtual reality in the Management of Football Injuries. Medicina 60:1000. doi: 10.3390/medicina60061000, 38929617 PMC11205647

[ref9007] Edwards A. Hettinga F. (2023). Virtual reality exercise platforms and the possibility for novel, engaging research in sport, exercise and health. Perform. Enhanc. Health. doi: 10.1016/j.peh.2023.100253 PMC10209421 37251496

[ref13] Fiore M. Bianconi A. Sicari G. Conni A. Lenzi J. Tomaiuolo G. . (2024). The use of smart rings in health monitoring—a meta-analysis. Appl. Sci. 14:10778. doi: 10.3390/app142310778

[ref14] Guneralp H. Yavuz H. U. Sekeroglu B. Oytun M. Tinazci C. (2025). Analysis of combined strength training with small-sided games in football education using machine learning methods. Appl. Sci. 15:5672. doi: 10.3390/app15105672

[ref15] Ju C.-Y. Kim J.-H. Lee D.-H. (2023). GolfMate: enhanced golf swing analysis tool through pose refinement network and explainable golf swing embedding for self-training. Appl. Sci. 13:11227. doi: 10.3390/app132011227

[ref16] Jud M. Thalmann S. (2024). AI in digital sports coaching – a systematic review. Manag. Sport Leis. 30, 1577–1593. doi: 10.1080/23750472.2024.2449016

[ref17] Kadhim E. J. Almayah S. K. Aldewan L. H. M. Ghazi M. A. (2025). Developing an artificial intelligence system to analyse and evaluate performance technical kata movements in karate | scientific journal of sport and performance. Available online at: https://sjsp.aearedo.es/index.php/sjsp/article/view/ai-and-performance-technical-kata-movements-in-karate (accessed September 11, 2025).

[ref18] Kalén A. Rey E. de Sal Rellán-Guerra A. Lago-Peñas C. (2019). Are soccer players older now than before? Aging trends and market value in the last three decades of the UEFA champions league. Front. Psychol. 10:76. doi: 10.3389/fpsyg.2019.00076 30745889 PMC6360147

[ref19] Karabıyık H. Tugay D. (2023). Analyzing collective behaviours in FIFA world cup Qatar 2022. SPORMETRE Beden Eğitimi ve Spor Bilimleri Dergisi 21, 226–236. doi: 10.33689/spormetre.1249071

[ref20] Keskar A. G. Bhurchandi K. M. Kamble P. R. (2019). A deep learning ball tracking system in soccer videos. Opto-Electron. Rev. 27, 58–69. doi: 10.1016/j.opelre.2019.02.003

[ref21] Kittel A. Larkin P. Elsworthy N. Spittle M. (2019). Using 360° virtual reality as a decision-making assessment tool in sport. J. Sci. Med. Sport 22, 1049–1053. doi: 10.1016/j.jsams.2019.03.012, 30987883

[ref22] Li H. Zhang M. (2021). Artificial intelligence and neural network-based shooting accuracy prediction analysis in basketball. Mob. Inf. Syst. 2021:4485589. doi: 10.1155/2021/4485589

[ref23] Lian D. (2024). Deep learning in sports skill learning: a case study and performance evaluation. EAI Endorsed Trans. Pervasive Health Technol. 10:5809. doi: 10.4108/eetpht.10.5809

[ref24] Liu Z. (2024). Visualization of basketball tactical evolution in generative AI big models for teaching and learning. Appl. Math. Nonlinear Sci. 9:20241915. doi: 10.2478/amns-2024-1915

[ref25] Mascret N. Montagne G. Devrièse-Sence A. Vu A. Kulpa R. (2022). Acceptance by athletes of a virtual reality head-mounted display intended to enhance sport performance. Psychol. Sport Exerc. 61:102201. doi: 10.1016/j.psychsport.2022.102201

[ref26] Medina-Ramírez R. Mallol Soler M. García F. Pla F. Báez-Suárez A. Teruel Hernández E. . (2024). Effects in sleep and recovery processes of NESA neuromodulation technique application in young professional basketball players: a preliminary study. Stress 4, 238–250. doi: 10.3390/stresses4020014

[ref27] Moustakas L. (2025). Game changer: harnessing artificial intelligence in sport for development. Soc. Sci. 14:174. doi: 10.3390/socsci14030174

[ref28] Naik B. T. Hashmi M. F. Geem Z. W. Bokde N. D. (2022). DeepPlayer-track: player and referee tracking with Jersey color recognition in soccer. IEEE Access 10, 32494–32509. doi: 10.1109/ACCESS.2022.3161441

[ref29] Nambi G. Abdelbasset W. Elsayed S. Alrawaili S. Abodonya A. Saleh A. . (2020). Comparative effects of isokinetic training and virtual reality training on sports performances in university football players with chronic low back pain-randomized controlled study. Evid. Based Complement. Alternat. Med. 2020:2981273. doi: 10.1155/2020/2981273 32617104 PMC7315304

[ref30] Pastel S. Petri K. Chen C. H. Wiegand Cáceres A. M. Stirnatis M. Nübel C. . (2023). Training in virtual reality enables learning of a complex sports movement. Virtual Reality 27, 523–540. doi: 10.1007/s10055-022-00679-7

[ref31] Peña-González I. Henríquez M. Sarabia J. M. Moya-Ramón M. (2024). Age does not influence the physical performance of football players with cerebral palsy. Sci. Sports 39, 377–383. doi: 10.1016/j.scispo.2023.10.003

[ref32] Postupaiev S. Damaševičius R. Maskeliūnas R. (2024). Real-time camera operator segmentation with YOLOv8 in football video broadcasts. AI 5, 842–872. doi: 10.3390/ai5020042

[ref33] Puce L. Bragazzi N. L. Currà A. Trompetto C. (2025). Harnessing generative artificial intelligence for exercise and training prescription: applications and implications in sports and physical activity—a systematic literature review. Appl. Sci. 15:3497. doi: 10.3390/app15073497

[ref34] Rahimian P. Toka L. (2022). Optical tracking in team sports: a survey on player and ball tracking methods in soccer and other team sports. J. Quant. Anal. Sports. 18, 35–57. doi: 10.1515/jqas-2020-0088

[ref35] Rusmanto R. Tomoliyus T. Sulastion A. Gazali N. Abdullah K. H. Gil-Espinosa F. J. . (2023). Virtual reality to promoting sports engagement and some technical skills in junior football athletes: a 12-week randomized controlled trial. Retos 50, 1129–1133. doi: 10.47197/retos.v50.100319

[ref36] Sun W. (2022). Predictive analysis and simulation of college sports performance fused with adaptive federated deep learning algorithm. J Sens 2022:1205622. doi: 10.1155/2022/1205622

[ref37] Takamidoa R. Suzukia C. Nakamoto H. (2025). Personalized Motion Guidance Framework for Athlete-Centric Coaching. arXiv. doi: 10.48550/arXiv.2510.10496

[ref38] Tan J. Chen J. (2025). Generating context-specific sports training plans by combining generative adversarial networks. PLoS One 20:e0318321. doi: 10.1371/journal.pone.0318321, 39883653 PMC11781690

[ref39] Tondut J. Dandrieux P. Caumeil B. Ruffault A. Giroux C. Guilhem G. . (2023). Estimation du risque de blessures en utilisant le *machine learning* basée sur le *monitoring* de la perception des états physiques et mentaux des athlètes: étude préliminaire sur 110 athlètes de haut niveau suivis sur une période de 18 mois. J. Traumatol. Sport. 40, 74–80. doi: 10.1016/j.jts.2023.04.002

[ref40] Tooth C. Kaux J. -F. Leclerc S. (2024). Épidémiologie des commotions cérébrales dans le sport. J. Traumatologie Sport Commotion Cérébrale 41, 200–204. doi: 10.1016/j.jts.2024.06.002

[ref42] Van Eetvelde H. Mendonça L. D. Ley C. Seil R. Tischer T. (2021). Machine learning methods in sport injury prediction and prevention: a systematic review. J. Exp. Orthop. 8:27. doi: 10.1186/s40634-021-00346-x, 33855647 PMC8046881

[ref43] Wang Y. Liu W. Liu X. (2022). Explainable AI techniques with application to NBA gameplay prediction. Neurocomputing 483, 59–71. doi: 10.1016/j.neucom.2022.01.098

[ref44] Wang Z. Veličković P. Hennes D. Tomašev N. Prince L. Kaisers M. . (2024). TacticAI: an AI assistant for football tactics. Nat. Commun. 15:1906. doi: 10.1038/s41467-024-45965-x, 38503774 PMC10951310

[ref45] Wesely S. Hofer E. Curth R. Paryani S. Mills N. Ueberschär O. . (2025). Artificial intelligence for objective assessment of acrobatic movements: applying machine learning for identifying tumbling elements in cheer sports. Sensors 25:2260. doi: 10.3390/s25072260, 40218772 PMC11991202

[ref46] Westerbeek H. (2025). Algorithmic fandom: how generative AI is reshaping sports marketing, fan engagement, and the integrity of sport. Front. Sports Act. Living 7:1597444. doi: 10.3389/fspor.2025.1597444, 40406423 PMC12094936

[ref47] Westerbeek H. Van Schaik T. (2025). Platform power, athlete branding, generative AI, and the future of sport governance—a systematic review. Front. Sports Act. Living 7:1642180. doi: 10.3389/fspor.2025.1642180, 41031328 PMC12477720

[ref48] Xu T. Tang L. (2021). Adoption of machine learning algorithm-based intelligent basketball training robot in athlete injury prevention. Front. Neurorobot. 14:620378. doi: 10.3389/fnbot.2020.620378, 33519414 PMC7843384

[ref49] Yi Q. Groom R. Dai C. Liu H. Gómez Ruano M. Á. (2019). Differences in technical performance of players from ‘the big five’ European football leagues in the UEFA champions league. Front. Psychol. 10:2738. doi: 10.3389/fpsyg.2019.02738, 31866914 PMC6908525

[ref50] Zhao K. Xueying Guo (2022). Analysis of the application of virtual reality technology in football training. J Sens:1339434. doi: 10.1155/2022/1339434

[ref51] Zouhal H. Coppalle S. Ravé G. Dupont G. Jan J. Tourny C. . (2021). Football de haut-niveau: analyses physique et physiologique – blessures et prévention. Sci. Sports 36, 332–357. doi: 10.1016/j.scispo.2021.03.001

